# School polices, programmes and facilities, and objectively measured sedentary time, LPA and MVPA: associations in secondary school and over the transition from primary to secondary school

**DOI:** 10.1186/s12966-016-0378-6

**Published:** 2016-04-26

**Authors:** Katie L. Morton, Kirsten Corder, Marc Suhrcke, Flo Harrison, Andy P. Jones, Esther M. F. van Sluijs, Andrew J. Atkin

**Affiliations:** UKCRC Centre for Diet and Activity Research (CEDAR), MRC Epidemiology Unit, University of Cambridge School of Clinical Medicine, Box 285 Institute of Metabolic Science, Cambridge Biomedical Campus, Cambridge, CB20QQ UK; Centre for Health Economics, University of York, York, UK; UKCRC Centre for Diet and Activity Research (CEDAR) and Norwich Medical School, University of East Anglia, Norwich, UK

**Keywords:** Physical activity, Sedentary behaviour, Adolescent, School environment, School transition

## Abstract

**Background:**

There is increasing policy interest in ensuring that the school environment supports healthy behaviours. We examined the cross-sectional and longitudinal associations between schools’ policies, programmes and facilities for physical activity (PA) and adolescents’ objectively-measured activity intensity during the school day and lunchtime.

**Methods:**

Accelerometer-derived PA (proportion of time spent in sedentary (SED), light PA (LPA) and moderate-to-vigorous PA (MVPA)) during school hours and lunchtime from 325 participants in the SPEEDY study were obtained from baseline measurements (primary school, age 9/10 years) and +4y follow-up (secondary school). School environment characteristics were assessed by teacher questionnaire. Multivariable multi-level linear regression analyses accounting for school and adjusted for sex, age, BMI and family socio-economic status assessed cross-sectional associations with lunchtime and school-day SED, LPA and MVPA; effect modification by sex was investigated. The association of changes in school environment with changes in outcomes was examined using multivariable cross-classified linear regression models.

**Results:**

There were significant differences between primary and secondary schools for 6/10 school environment characteristics investigated (including secondary schools reporting shorter breaks, more lunchtime PA opportunities, and higher number of sports facilities). Cross-sectional analyses showed that boys attending secondary schools with longer breaks spent significantly less time in SED and more time in MVPA during the school day. Longitudinally, an increase in break-time duration between primary and secondary school was associated with smaller reductions in MVPA during the school day. Moreover, participants who moved from a primary school that did not provide opportunities for PA at lunchtime to a secondary school that did provide such opportunities exhibited smaller increases in SED and smaller reductions in MVPA at lunchtime.

**Conclusions:**

Schools should consider the potential negative impact of reducing break time duration on students’ MVPA and SED during the school day. School-based interventions that combine longer breaks and more PA opportunities during lunchtime may be a fruitful direction for future research. Further research should also explore other factors in the school environment to explain the school-level clustering observed, and study sex differences in the way that the school environment influences activity intensity for adolescent populations.

**Electronic supplementary material:**

The online version of this article (doi:10.1186/s12966-016-0378-6) contains supplementary material, which is available to authorized users.

## Background

The promotion of physical activity among young people is a public health priority [1]. Data from several countries suggest that a substantial proportion of children are insufficiently active to achieve health benefit [2, 3], and that levels of physical activity decline throughout childhood and into adolescence [4, 5]. Furthermore, sedentary behaviour may have detrimental health effects in young people, independent of the level of physical activity acquired [6], as well as its own unique correlates [7].

Schools are considered an important setting for the promotion of health and well-being in young people, and there is increasing policy interest in ensuring that the school environment supports activity [8–10]. A growing number of studies have explored how features of the school’s environment impact upon activity. For example, characteristics of the physical environment, such as better outdoor facilities [11] and larger school campuses and play areas [12], have shown associations with greater moderate-to-vigorous physical activity (MVPA). However, the evidence for the influence of the physical environment (for adolescent populations especially) is generally mixed [13, 14]. For example, in a previous analysis of SPEEDY [15] data, Harrison and colleagues identified few associations between objectively measured features of the school physical environment and adolescent MVPA [14]. The current study extends this research by considering how multiple school-related factors, beyond the objectively measured *physical* environment, may be associated with adolescent physical activity and sedentary time. This includes “whole-school” policies, programmes and resources for physical activity.

The majority of studies that have explored the school policy environment for physical activity have been undertaken in primary schools [16–18], limiting what can be applied to older students in secondary schools. Several studies have shown that the *transition* from primary to secondary school is marked by a change in activity amount and pattern, with the direction and exact nature of the change seemingly attributable, at least in part, to features of the school environment (e.g., changes in policies, programmes and facilities for physical activity) [4, 19]. A recent study examined the changes in the school environment from primary to secondary school and found that secondary schools were more likely to foster activity during school hours, whereas primary schools were more likely to promote physical activity after-school [19]. Specifically, secondary schools scored more positively on school environment characteristics (e.g., active schoolyards and playgrounds and health education policy) but lower on sport and physical activity after-school. These findings are noteworthy given that other studies have shown that physical activity in school hours decreases during the transition to secondary school (especially during lunch times; [20]). How changes in the school environment between primary and secondary school affect students’ activity behaviour is however largely unknown. Moreover, most research has focused on MVPA and not considered light physical activity and/or sedentary behaviour.

This study aims to add to the limited knowledge on the importance of the secondary school environment for student activity intensity (sedentary time (SED), light physical activity (LPA, and MVPA). The specific objectives are; (a) to explore changes in the physical activity supportiveness of school policies, programmes and facilities between primary and secondary schools, (b) to assess the associations between secondary school policies, practices and facilities for physical activity and objectively measured *adolescent* activity intensity during the school day and at lunchtime, and (c) to examine the longitudinal association between change in school physical activity policies, practices and facilities and change in objectively measured activity intensity during the school day and at lunchtime.

## Methods

### Recruitment and data collection

The SPEEDY (Sport, Physical activity and Eating behaviour: Environmental Determinants in Young people) study is a population based longitudinal cohort study which sought to investigate the factors associated with diet and physical activity behaviour of young people in the county of Norfolk, England. Methods of school and participant recruitment and data collection procedures have been described in detail elsewhere [15, 21] therefore only a brief overview is provided below, focusing on the specific measures utilised in the current analyses.

In 2007, 157 primary schools across the county of Norfolk with at least 12 Year 5 pupils (age 9/10 years) were sampled according to stratification by urban/rural status. Ninety two primary schools were recruited. Baseline data collection was performed (*n* = 2064) during the school summer term (April to July 2007; ‘SPEEDY 1’). Child height and weight were measured by trained researchers and used to calculate body mass index (BMI, in kg/m^2^). Parent’s self-reported highest level of education, home ownership, car ownership and ethnicity were obtained from a questionnaire survey.

Participants with an active postal address and who had not withdrawn from the study were contacted via their home address 4 years later, when aged 13/14y (school year 9; the third year of secondary education). Follow-up assessment was undertaken during the school summer term of 2011 (‘SPEEDY 3’), in which all covariates mentioned above were measured again using the same methods [20, 21].

### Measurement of activity intensity

At both time points, time spent in activity intensities was measured objectively using an Actigraph (GT1M; Pensacola, FL) accelerometer, set to record at 5-s epochs [22]. The Actigraph has been shown to accurately assess energy expenditure among European children and adolescents during free-living conditions [23, 24]. Participants were instructed to wear the monitor during waking hours for 7 days and to remove it while bathing, showering and swimming.

Accelerometer data were analysed using a batch processing programme (http://www.mrc-epid.cam.ac.uk/research/resources/). Periods of ≥10 min of continuous zeros were considered non-wear time and excluded [25, 26]. Thresholds for defining activity intensities (scaled to 5-s epochs) were as follows: sedentary time (SED) <100 cpm (cpm), light physical activity (LPA) ≥101–1999 cpm [27], MVPA ≥2000 cpm. A lower threshold of 2000 cpm to define MVPA has been used previously in this study [28] and others [26] and is equivalent to walking at 4 km/h [23].

Based on school-reported start and end times of the school day and lunch time break, we derived the duration of these periods at the school level and matched them minute-by-minute with the accelerometry data. The primary outcome variables were expressed as the proportion of accelerometer wear time (cross-sectional analysis) or *change* in the proportion of accelerometer wear time (longitudinal analysis) in each activity intensity (SED, LPA, MVPA), accounting for differences in duration of break and school day. Outcome variables were derived separately for lunch time and the whole school day. A valid observation was defined as wear time ≥80 % of the duration of lunch time or the school day. For each time period, a minimum of 2 valid days of observation was required for inclusion in the analysis.

### School-level variables

At both SPEEDY 1 and SPEEDY 3, a questionnaire asking about the school policies, programmes and facilities was distributed to school head teachers [18]. Table 1 describes the variables assessed. All 92 primary schools at SPEEDY 1 returned the questionnaire. Students who participated in SPEEDY 1 (and agreed to follow-up in SPEEDY 3) attended 49 different secondary schools. Schools with < 2 students from the SPEEDY study in attendance were not asked to complete a school questionnaire (related to *N* = 8 participants). This left 43 secondary schools at SPEEDY 3 that returned the school questionnaire.

**Table 1:** Description, descriptive characteristics, and differences between primary and secondary schools for school questionnaire-based data collected

Variable	Assessment	Primary schools	Secondary schools	P for difference
		Proportion/mean (SD)	Proportion/mean (SD)	
Length of break *(minutes per day)*	Morning break, lunch break (and any other breaks, e.g., pm) duration added to obtain total break time.	75.21 (8.28)	65.70 (10.67)	.000**
Number of sports facilities *(medium/high quality)*	Reported access to nine sport related facilities (e.g., gym, swimming pool etc.). Those rated as high or radium quality were summed.	5.30 (1.30)	6.35 (1.09)	.027*
Hours of PE *(per week)*	Reported and rounded to the nearest ½ hour.	2.09 (.36)	2.24 (.412)	.241
Physical activity policy *(% yes)*	“Does your school have a policy to promote physical activity” (written or informal)	84.8	79.1	.464
Lunchtime extra-curricular physical activity provision *(% yes)*	“Does your school provide any extracurricular physical activity during lunch breaks?”	62	88.1	.005*
School attitude (to physical activity promotion)	Reported agreement with five statements about school attitude to physical activity (5-point Likert scale). Scores were summed and averaged.	4.44 (.92)	4.39 (.69)	.749
Compulsory outdoor break, if weather allows it *(% yes)*	Choosing one of five options reflecting outdoor policy during break times. The answers were collapsed into a dichotomous category.	96.7	27.9	.000**
Activity rules during breaks:	Report of whether children are allowed to do screen-based activity (e.g., watch TV or use computers) regardless of weather.	8.7	74.4	.000**
*- Screen-based activity allowed (% yes)*				
*-Physically-active activities allowed (% ≥ 2)*	Report of whether children are allowed to do physically active activities (e.g., use sports equipment, play ball games, play running games etc.) regardless of the weather	52.2	65	.194
School Environment (physical environment)	Reported agreement with seven statements about the area around the school (5-point Likert scale). Scores were summed (possible score 0–35)	22.01 (3.96)	24.91 (3.34)	.001*

*PA* physical activity, *SD* Standard deviation; * = *p* < .005; ** = *p* < .001

### Covariates

The child’s sex, age (at SPEEDY 3), BMI (at SPEEDY 3) and family SES (at SPEEDY 1) were included in analytical models as covariates. Family SES was measured as a score calculated as the sum of three variables; parent-reported age at leaving full time education (≤16 years coded 0; >16 years coded 1), car ownership (no coded 0; yes coded 1), and house ownership (rental coded 0; own/buying coded 1). Participants were assigned to low (score 0/1), mid (score 2) or high (score 3) SES groups as used in a previous study [29].

### Data analyses

All analyses were undertaken using STATA version 13 (Stata, College Station, TX). Three separate analyses were undertaken to address the objectives outlined above.

#### Changes in the school environment for PA from primary to secondary school

Differences in the school environment between the primary schools and secondary schools were tested using independent samples t-tests and chi-square tests.

#### Cross-sectional associations at secondary school

Outcome variables for SED and LPA were normally distributed. MVPA (school-day and lunch time) was not normally distributed, with a small number of extreme outliers; therefore we curtailed outliers to the 95^th^ percentile value to achieve acceptable levels of kurtosis. Multivariable multi-level linear regression was used to assess cross-sectional associations between school policies, programmes, and facilities and activity intensity, accounting for school-level clustering and adjusted for sex, age, BMI and family SES. As previous evidence suggests that features of the school environment impact boys and girls differently [30], interactions with sex were explored.

First, a null-model was created to estimate school-level variance for each outcome, adjusted for all covariates. Second, the association between each exposure variable and each outcome variable was assessed; variables with a P-value of less than .25 were retained at this stage in order to minimise Type 2 error as a result of confounding amongst the exposure variables. Third, interactions between each exposure variable and sex were assessed for each outcome variable. Interactions were retained for inclusion in the multivariable model where they met the following criteria: 1) the interaction term P-value was <0.1 and 2) the determinant was associated at *P* < 0.05 for at least one of the sexes in a stratified model. This strategy was employed to simplify interpretation of the final multivariable model and to reduce the risk of type 1 error resulting from multiple hypothesis testing. Lastly, the final models were developed, including only those variables and interactions terms retained from steps 2 and 3 described above. This model building strategy was also applied in the longitudinal analyses.

#### Longitudinal analyses (primary to secondary school)

To account for differences in school/lunch duration at primary and secondary school, outcomes were derived as change in the proportion of wear-time spent in each intensity category (SED, LPA, MVPA) from primary to secondary school. Outcome variables for change in SED and LPA were normally distributed. Change in MVPA (school-day and lunch time) was not normally distributed, due to a small number of extreme outliers; these values were curtailed at the 99^th^ percentile for use in the analysis.

For continuous exposure variables (e.g., hours of PE, length of break duration etc.) change variables were derived as baseline subtracted from follow-up. Change in PE duration was categorised as ‘no change’, ‘decrease’ or ‘increase’. Categorical variables were derived to reflect changes in binary exposures from baseline to follow-up (e.g., “does the school have a physical activity policy?” was coded into ‘no/no’, ‘no/yes’, ‘yes/yes’ and ‘yes/no’ reflecting all four possible options). Where individual categories contained less than 5 % of responses these were collapsed with other categories where appropriate. For example, the physical activity policy variable contained only12 cases in the ‘no/no’ group. Therefore this was combined with the ‘yes/yes’ group to create one reference group that reflected ‘no change’ in physical activity policy. For exposures relating to the requirement to be outdoors during break times and the use of screens at break time, derivation of change variables resulted in one category that comprised just one observation. These categories were recoded to missing and not estimated in regression models.

The association of changes in school environment with changes in SED, LPA and MPVA was examined using cross-classified multi-level linear regression models. The cross-classified model accounts for the clustering of participants within primary and secondary schools but does not assume a hierarchical structure as children from any given primary school attended several different secondary schools, and each secondary school received pupils from several different primary schools.

As described for the cross-sectional analyses, simple models were developed to look at associations between each exposure (change) variable and each outcome (change) variable. Then interactions with sex were explored. Finally, full models were developed with those variables retained from the initial steps.

## Results

There were no differences in the age, sex or BMI (all: *p* > 0.05) of participants that provided valid accelerometer data for either lunch time or the whole school day at SPEEDY3 relative to those that were assessed at SPEEDY 1. Characteristics of participants who provided valid accelerometer data at both SPEEDY 1 and SPEEDY 3 are shown in Table 2, with participants contributing an average of 4.2 to 4.7 valid days.

**Table 2:** Sample characteristics for SPEEDY participants included in these analyses (*n* = 325)

	SPEEDY 1 (2007)		SPEEDY 3 (2011)	
Sex (%)				
Male	47.7		-	
Female	52.3		-	
Age (years, mean(SD))	10.24 (.32)		14.32 (.30)	
Ethnicity (%)				
White	97		-	
Other	3		-	
BMI (mean(SD))	18.05 (3.15)		20.95 (4.01)	
Family SES (%)				
Low	18.3		-	
Middle	39.1		-	
High	42.6		-	
Physical activity	School day	Lunchtime	School day	Lunchtime
	(*n* = 321)	(*n* = 321)	(*n* = 315)	(*n* = 325)
No. of valid days per participant (mean(SD))	4.3 (0.8)	4.7 (0.7)	4.2 (0.9)	4.3 (0.9)
Proportion of wear-time (%)				
SED	71	53	77	64
Light PA	21	29	16	23
MVPA	8	18	7	13
Time spent (minutes, mean(SD))				
SED	272.90 (25.54)	31.27 (7.00)	300.16 (29.87)	31.07 (9.59)
LPA	80.65 (16.31)	17.00 (3.50)	64.65 (18.87)	11.33 (4.60)
MVPA	30.76 (10.76)	10.55 (4.92)	27.43 (13.70)	6.49 (4.66)

SD, standard deviation; SED, sedentary behaviour; MVPA, moderate to vigorous intensity physical activity. Valid days were defined as days in which wear time ≥80 % of the duration of lunchtime and/or school day

### Differences in the school-environment between primary and secondary school

Table 1 shows the exposure variable scores for the primary and secondary school samples. Secondary schools had shorter break time, a higher number of good quality sports facilities, a more positive perceived physical environment (for promoting physical activity) surrounding the school, and greater provision of lunchtime extracurricular physical activity compared to primary schools. In relation to school-based break time policies, a greater proportion of secondary schools allowed screen-based activities during break times. In addition, a significant difference was observed for break time policy. Specifically, more primary schools had a rule that pupils must go outdoors if the weather is dry.

### Cross-sectional associations between the school environment and adolescent activity intensity

We analysed data from 325 participants at SPEEDY 3 who provided valid accelerometer data. Intraclass correlation coefficients indicated that school-level differences accounted for 16 %, 12 % and 15 % of the variance in *lunchtime* SED, LPA and MVPA respectively, and 7 %, 2 %, and 23 % of the variance in *school-day* SED, LPA and MVPA respectively.

Based on the simple models, two variables were taken forwards for *lunchtime* SED and MVPA models (hours of PE, and compulsory outdoor break) and three variables for the *school-day* models (hours of PE, length of break time and school attitude). Additional files 1 and 2: Tables S1 and S2 show the results from the simple models for SED, LPA and MVPA during lunchtime and school-day respectively. No exposure variable was associated with LPA at *p* < 0.25. The final multivariable models are shown in Tables 3 and 4 (*lunchtime* and *school-day*, respectively). None of the variables were associated at *p* < 0.05.

**Table 3:** Cross-sectional association of school policies, practices and facilities with adolescent sedentary time and physical activity during lunchtime (multivariable models)

Exposure	MVPA (*n* = 276)	
	β	(95 % CI)
Hours of PE	0.015	(−.000, .030)
Compulsory outdoor break	0.019	(−.010, .048)

Co-efficient represents proportion of wear time in MVPA

**Table 4:** Cross sectional association of school policies, practices and facilities with adolescent sedentary time and physical activity during the whole school day (multivariable models)

Exposure	SED (*n* = 268)		MVPA (*n* = 268)	
	β	(95 % CI)	β	(95 % CI)
Length of break	−0.001	(−.002, .000)	.000	(−.000, .001)
Hours of PE	−0.010	(−.022, .003)	.006	(−.000, .001)
School attitude	−0.011	(−.024, .002)	N/A	

Co-efficient represents proportion of wear time in SED and MVPA. N/A, not analysed in multiple model

### Sex differences in the cross-sectional associations

Three interactions with sex were explored further in the final models. There was evidence of effect modification by sex for the association between total duration of break and school-day SED (β for interaction = −0.0013, *p* = .098). Subgroup analyses showed that boys attending schools with longer breaks spent a significantly lower proportion of their wear time in SED (β = −0.0013, *p* = .024). This equates to approximately 20 min across the school day when comparing the SPEEDY secondary schools with the longest (95 mins) and shortest break duration (50 mins). There was no association for girls (β = −0.000039, *p* = .995). A second interaction with total duration of break was identified for school-day MVPA (β = −0.00070, *p* = .040). Subgroup analyses showed that boys attending schools with longer breaks spent a greater proportion of wear time in MVPA (β = 0.00075, *p* = .018). This equates to approximately 12 min across the school day when comparing the SPEEDY secondary schools with the longest and shortest break duration. There was no association in girls (β = 0.000051, *p* = .882). Third, the association between break-time physical activity rules (i.e., physically active activities allowed) and school-day MVPA was also moderated by sex (β = −0.012, *p* = .051). Sex-stratified analyses revealed that the direction of association was in the opposite direction for boys and girls, however the associations were non-significant for both sexes (boys: β = 0.006, *p* = .397; girls: β = −0.006, *p* = .374).

### Longitudinal associations between changes in the school environment and changes in activity intensity

As reported in Table 2, and previously [21], lunchtime and school-day levels of MVPA and LPA decreased over the transition to secondary school, and both lunchtime and school-day SED increased. Additional files 3 and 4: Tables S3 and S4 show the results from the simple models for changes in SED, LPA and MVPA during lunchtime and school-day, respectively. Several school-level variables were taken forwards to the multivariable models, based on an association in the simple models, or evidence of a significant interaction (and sub-group) effect by sex.

Multivariable models for lunchtime and school-day are presented in Tables 5 and 6 respectively. Participants who moved from a primary school that did not provide extra-curricular physical activity at lunchtime to a secondary school that did provide such opportunities exhibited smaller increases in the proportion of monitor wear time spent sedentary during lunchtime and during the whole school day. They also exhibited smaller reductions in MVPA at lunchtime and during the school day. An increase in the duration of break time from primary to secondary school was associated with smaller reductions in MVPA across the whole school day. None of the examined exposures were associated with changes in LPA at *p* < 0.05 for lunchtime or school-day.

**Table 5:** Longitudinal association of changes in the school environment with changes in activity intensity during lunchtime (multivariable models)

Exposure	SED change (*n* = 293)		LPA change (*n* = 271)		MVPA change (*n* = 267)	
	β	(95 % CI)	β	(95 % CI)	β	(95 % CI)
School environment	N/A		N/A		(−0.25, 0.24)	
Length of break	0.10	(−0.09, 0.28)	−0.036	(−0.15, 0.08)	N/A	
Hours of PE						
no change (reference)	N/A		N/A			
decrease					−1.46	(−4.42, 1.49)
increase					0.80	(−2.01, 3.60)
Physical activity policy						
no change (reference)						
no/yes	−2.26	(−7.51, 3.00)	1.36	(−1.97, 4.70)	0.33	(−2.63, 3.28)
yes/no	1.89	(−3.66, 7.43)	−0.28	(−3.54, 3.00)	−1.06	(−4.02, 1.91)
Lunchtime extracurricular physical activity provision						
no change (reference)						
no/yes	**−5.32**	(−9.03, −1.61)**	2.40	(−0.07, 4.88)	**3.16**	(1.09, 5.23)**
yes/no	−7.00	(−15.41, 1.42)	4.63	(−0.52, 9.77)	2.71	(−1.60, 7.02)
Break time rule: screen-use						
no/no (reference)	N/A				N/A	
no/yes			−1.27	(−5.52, 2.98)		
yes/no			#	#		
yes/yes			−1.56	(−6.77, 3.66)		
Compulsory outdoor break rule						
no change (reference)						
no/yes	-	-	-	-	-	-
yes/no	4.77	(−0.32, 9.86)	−2.54	(−5.44, 0.36)	−1.63	(−4.54, 1.29)

LPA, light physical activity; MVPA, moderate to vigorous physical activity; PE, physical education; PA, physical activity; # = coefficient not estimated due to small cell size (*n* = 1); N/A, not analysed in multiple model

* = *p* < 0.05; ** = *p* < 0.01

**Table 6:** Longitudinal association of changes in the school environment with changes in activity intensity during the whole school day (multivariable models)

Exposure	SED change (*n* = 259)		LPA change (*n* = 269)		MVPA change (*n* = 259)	
	β	(95 % CI)	β	(95 % CI)	β	(95 % CI)
School attitude	N/A		−0.47	(−1.37, 0.43)	N/A	
School environment	N/A		0.07	(−0.04, 0.19)	−0.06	(−0.16, 0.04)
Length of break	−0.09	(−0.17, 0.00)	N/A		**0.06**	(0.02, 0.10)**
Hours of PE						
no change (reference)						
decrease	2.16	(−0.27, 4.60)	−1.41	(−2.94, 0.11)	−0.83	(−2.03, 0.37)
increase	0.88	(−1.35, 3.11)	−0.56	(−1.81, 0.70)	−0.09	(−1.27, 1.08)
Physical activity policy						
no change (reference)	N/A		N/A			
no/yes					0.12	(−1.19, 1.42)
yes/no					−0.54	(−1.81, 0.73)
Lunchtime extracurricular physical activity provision						
no change (reference)			N/A			
no/yes	−2.03	(−3.89, −0.17)*			**0.90**	(0.03, 1.77)*
yes/no	−1.10	(−4.89, 2.68)			0.07	(−1.88, 2.01)
Break time rules: physically active activities						
less/less (reference)					N/A	
less/more	2.16	(−0.84, 5.17)	−0.97	(−2.42, 0.47)		
more/less	0.81	(−2.28, 3.91)	−1.54	(−3.19, 0.11)		
more/more	1.07	(−1.76, 3.91)	−1.20	(−2.56, 0.16)		

*LPA* light physical activity, *MVPA* moderate to vigorous physical activity, *PE* physical education, *PA* physical activity, *N/A* not analysed in multiple model

* = *p* < 0.05; ** = *p* < 0.01

### Sex differences in the longitudinal associations

We examined the multivariable models with the inclusion of the interaction term taken forwards from the exploration of the simple models. For change in the proportion of wear time spent in LPA during the school-day, there was a significant interaction effect for sex for ‘PE duration increased’ (β = 2.42, *p* = .052). Boys who went to a secondary school with longer PE duration than primary school demonstrated larger decreases in school-day LPA (β = −1.842, *p* = .046) compared to those whose PE duration was unchanged. There was no association in girls for an increase in PE duration (from primary to secondary) and change in LPA during the school day (β = 0.57, *p* = .51). The sex interaction term for the association with lunchtime MVPA of moving from a school that was less permissive of physical activity to one that was more permissive was significant (β = −5.75, *p* = .03), but subgroups associations were non-significant (boys: β = 2.67, *p* = .30; girls: β = −3.07, *p* = .18). None of the other interactions from the simple models were significant in the multivariable models.

## Discussion

The analyses presented in this paper show that the supportiveness of the school environment for activity intensity differs between primary and secondary schools in Norfolk, UK. However, only total break duration and extra-curricular physical activity opportunities at lunchtime are associated with adolescent MVPA and SED.

This work shows that secondary schools generally have shorter break times, a higher number of good quality sports facilities, a more positive physical environment surrounding the school, and greater provision of lunchtime extracurricular physical activity compared to primary schools. De Meesters et al. [19] also found that secondary schools in their Belgian sample tended to score higher than primary schools in terms of numbers of physical activity facilities. A recent study also using data from the SPEEDY cohort, but using audit-based objective measures of the physical environment (SPEEDY audit tool [31]) found that primary schools scored more positively than secondary schools for their supportiveness of physical activity, and significantly so for sport and play facilities [14]. The questionnaire-based measures presented here asked schools to report the number of “medium and high quality” facilities at their school, which includes a subjective judgement about quality. In contrast, the audit tool [31] objectively assesses individual components relating to specific facilities for sport *and* play. In both the present study and in Harrison et al’s [14] study, there was no association with activity intensity for the number of quality facilities. However, this highlights the challenges of using different measures to assess the school’s physical environment, which should be considered in its interpretation and future studies.

Total duration of break times in secondary schools was associated with more MVPA and less SED in boys across the whole school day in cross-sectional analyses, whereas an increase in the duration of break time attenuated the reductions in MVPA between primary and secondary school in both sexes. A previous SPEEDY analysis also reported an association between the length of *morning break* and maintenance of MVPA in primary school children [32]. However, to our knowledge, this is the first study to explore the impact of break time duration on adolescent activity intensity.

The importance of break times within schools has been outlined in a number of reports over the last decade [33, 34]. Break times are becoming shorter, especially in secondary schools [34]. Within our study, there was a vast difference in the secondary school break arrangements with some secondary schools only receiving 50 min break in total throughout the school day, while others received up to 95 min of break throughout the school day. Given that for boys this difference can result in over 20 min less SED and 12 min more MVPA during the school day, these findings add to the body of evidence that supports the importance of protecting break times at schools.

In view of emerging evidence that sedentary behaviour may have independent health effects in young people [6], the findings of this study suggest that secondary schools should consider the potential impact of break time in order to foster health and well-being in students. Furthermore, increasing break time duration may also result in other social and educational benefits [33]. This is a topic worthy of further observational and intervention research. It may be that increasing break time length may provide a cost-effective way of reducing sedentary time in schools. However, it is unknown if there is any trade-off with educational attainment if classroom time is reduced to accommodate more break time. One might hypothesise that educational attainment would not be negatively impacted *if* the breaks are physically active in nature [35, 36], however this link might not arise from simply increasing break time duration alone. The acceptability and feasibility of this idea should be considered, with more research to understand why the association is not shown in girls. A recent qualitative study in primary schools found that boys and girls experiences of break times were different and called for interventions that focus on the wider *social* environment of break times [37] to optimise break times for boys *and* girls. A similar study in secondary schools would be useful to explore the differences in how boys and girls experience break times and what school policies and practices might improve the break time experience in female students, as increasing the length of break alone might not be sufficient. In our recent review of the literature [13], there was some qualitative evidence that suggested certain polices (such as open gyms during break times) appear to benefit boys more than girls. However, there were no quantitative studies that examined break time specific rules and regulations, let alone how these may be moderated by sex.

As previously reported by Corder et al. [21], our analyses showed that the transition from primary school to secondary school is associated with increases in SED, and decreases in LPA and MVPA (during the school day and during lunchtime specifically). This pattern is also shown in other Western countries [4]. The current analyses showed that an increase in the provision of extra-curricular physical activity at lunchtime between primary and secondary school attenuated these commonly observed changes. A recent systematic review of adolescent PA and SED, showed an indeterminate association for the provision of extra-curricular physical activity opportunities and adolescent physical activity – however, the included studies did not include measures for SED behaviour, nor did they specifically look at extra-curricular provision at *lunchtime* [13]. Given that this finding is similar for boys and girls, the provision of lunchtime extra-curricular physical activity opportunities should be considered a potential intervention strategy to maintain activity across the transition from primary to secondary school. Furthermore, combining the provision of extra-curricular physical activity opportunities with increasing the length of break duration might improve the break time experience for all young people in secondary schools. The feasibility of this approach however needs further exploration, as it may result in increased supervision requirements and the need to introduce more equipment and facilities. Furthermore, the specific activities that are offered should be considered in greater detail as it is likely that the choice of activities available would determine uptake (e.g., contact sports versus dance versus walking groups), along with the level of student involvement and choice in deciding what these activities will be [10, 38].

We also found that an increase in PE duration from primary to secondary school was associated with lower levels of LPA in boys only. This is an interesting observation and potentially reflects a compensation effect [39] (i.e., boys are reducing light PA due to an increase in activity in PE). No data was available on timing of PE classes and we are therefore unable to further explore this issue. It would however be interesting to probe this research question further, including an exploration of the reasons for the observed gender difference in this association.

A notable observation is the relatively high level of variance explained at the school-level for school-day MVPA. However, the majority of school policies, practices and facilities assessed in the present study are not associated with school-day MVPA. As previously mentioned, Harrison and colleagues [14] have conducted complementary analyses focusing on objective measures of the physical environment (school grounds scores) only. Their analyses found positive cross-sectional associations between active travel provision scores and commuting time MVPA for adolescent boys and those living further from school. However, there were very few associations between changes in school ground scores (between primary and secondary schools) and change in school-based MVPA. Taken together, this indicates that there are other aspects within the school environment that determine the supportiveness of the environment for MVPA across the school day that have not been picked up with either the audit instrument or questionnaire. This could for example include the quality of the PE teaching or other features of the social environment not assessed.

The school environment not only consists of the physical features of the school, but also the wider psycho-social characteristics, which includes school leadership and the wider school ethos surrounding physical activity and also individual teacher behaviours [13]. It is unlikely that these features are captured within our one item that assessed school ‘attitudes’ towards physical activity promotion. Future research should seek to utilise (or develop) better measures of the school’s social environment that capture constructs such as physical activity-related leadership and the wider ethos of the school relating to physical activity. There could be scope for developing more *objective* measures of these features – similar to an audit tool that could better assess policy and social environment related factors in schools.

### Strengths and limitations

We used a validated, objective measure of activity intensity, and followed up the same participants in order to assess change in behaviour in relation to change in school policies, programmes and facilities. Furthermore, individual school-level time segments for the school day (and lunch time) were incorporated to be able to explore precise effects on each time segment and reduce error in activity estimates. Finally, we also examined the associations of school policies, programmes and facilities and different activity intensities. This is important given the relative lack of research on secondary school students’ SED and LPA, compared to the abundance of research that focuses on MVPA [13].

Limitations should also be noted. Although the sample in SPEEDY 3 was representative of those included in the SPEEDY 1 sample within the current analyses, this overall sample represented 15 % of the original SPEEDY sample (*n* = 2064), which itself contained a higher proportion of girls, and a lower proportion of obese children than the Norfolk population [15]. In addition, the Norfolk population, and hence our sample, is largely white, potentially making these results less relevant to other populations. The analytical sample size may have led to Type 2 errors although our analytical approach was designed to take this into consideration. In relation to our exposure measures, as outlined in the section above, a limitation is the relatively narrow focus of the school environment measure.

## Conclusions

To conclude, we observed differences in school policies, programmes and facilities for physical activity between primary and secondary schools in Norfolk, UK. However, the findings indicate that only two of the school-level variables assessed, total break duration and extra-curricular physical activity opportunities at lunchtime, are associated with adolescent MVPA and SED. These findings support the development of interventions to promote physical activity and reduce sedentary behaviour in adolescents that focus on school break duration and the provision of extra-curricular activities at lunchtime. Further research should explore other factors in the school environment, especially features of the social environment, in order to explain the school-level clustering observed and inform intervention development.

### Ethics approval

University of East Anglia Faculty of Health Ethics Committee.

## Additional files

Additional file 1: Table S1. Simple models; Cross-sectional association of school policies, programmes and facilities and adolescent activity intensity during lunchtime. (DOC 41 kb) Additional file 2: Table S2. Simple models; Cross-sectional association of school policies, programmes and facilities and adolescent activity intensity during the school day. (DOC 42 kb) Additional file 3: Table S3. Simple models; Association of changes in the school environment with changes in activity intensity during the whole school day. (DOC 45 kb) Additional file 4: Table S4. Simple models; Association of changes in the school environment with changes in activity intensity during lunchtime. (DOC 44 kb)
