# Supplementary material for: School polices, programmes and facilities, and objectively measured sedentary time, LPA and MVPA: associations in secondary school and over the transition from primary to secondary school
Source: Int J Behav Nutr Phys Act. 2016 Apr 26;13:54. doi: 10.1186/s12966-016-0378-6 (PMC4845338; doi:10.1186/s12966-016-0378-6)
Supplement: Additional file 1: Table S1. — Simple models; Cross-sectional association of school policies, programmes and facilities and adolescent activity intensity during lunchtime. (DOC 41 kb) [file 12966_2016_378_MOESM1_ESM.doc]

**Supplemental Table 1. Simple models; Cross-sectional association of school policies, programmes and facilities and adolescent activity intensity** during lunchtime.

| Exposure | SED | | LPA | | MVPA | |
| --- | --- | --- | --- | --- | --- | --- |
|  | β | (95% CI) | β | (95% CI) | 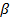 | (95% CI) |
| Length of break (minutes) | 0.000 | (-.002, .003) | -0.000 | (-.002, .001) | -0.000 | (-.001, .001) |
| Number of high quality facilities | 0.003 | (-.013, .018) | -0.002 | (-.010, .006) | -0.001 | (-.009, .007) |
| Hours of PE | -0.016 | (-.048, .016) | 0.001 | (-.018, .019) | **0.015** | **(-.002, .032)** |
| Physical activity policy | 0.005 | (-.051, .061) | -0.011 | (-.041, .020) | 0.007 | (-.022, .037) |
| Provision of extra-curricular lunchtime physical activity | -0.004 | (-.089, .081) | 0.001 | (-.045, .048) | 0.002 | (-.432, .047) |
| School attitude | -0.019 | (-.052, .015) | 0.008 | (-.010, .027) | 0.011 | (-.007, .029) |
| Compulsory outdoor break (in good weather) | -0.046 | (-.103, .011) | 0.029 | (-.002, .060) | **0.018** | **(-.013, .049)** |
| Break time rules: screen use allowed | -0.003 | (-.074, .067) | -0.000 | (-.039, .039) | 0.002 | (-.036, .040) |
| Break time rules: physically active activities allowed | -0.004 | (-.054, .047) | -0.007 | (-.034, .020) | 0.008 | (-.018, .035) |
| School (physical) environment | 0.001 | (-.007, .009) | -0.001 | (-.005, .004) | -0.001 | (-.005, .004) |

All exposure variables controlled for sex, age, BMI, and family SES; co-efficients shown in **bold** were taken forwards to multivariable models (p < .25).
