# Supplementary material for: School polices, programmes and facilities, and objectively measured sedentary time, LPA and MVPA: associations in secondary school and over the transition from primary to secondary school
Source: Int J Behav Nutr Phys Act. 2016 Apr 26;13:54. doi: 10.1186/s12966-016-0378-6 (PMC4845338; doi:10.1186/s12966-016-0378-6)
Supplement: Additional file 2: Table S2. — Simple models; Cross-sectional association of school policies, programmes and facilities and adolescent activity intensity during the school day. (DOC 42 kb) [file 12966_2016_378_MOESM2_ESM.doc]

**Supplemental Table 2. Simple models; Cross-sectional association of school policies, programmes and facilities and adolescent activity intensity during the school** day.

| Exposure | SED | | LPA | | MVPA | |
| --- | --- | --- | --- | --- | --- | --- |
|  | β | (95% CI) | β | (95% CI) | 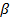 | (95% CI) |
| Length of break (minutes) | **-0.001** ^*^ | **(-.002, .000)** | **0.003^ᴛ^** | **(-.000, .001)** | **0.001*** | **(-.000, .001)** |
| Number of high quality facilities | -0.002 | (-.007, .004) | -0.000 | (-.003, .003) | 0.002 | (-.002, .006) |
| Hours of PE | **-0.013** | **(-.026, -.000)** | 0.004 | (-.004, .012) | **0.008** | **(.001, .015)** |
| Physical activity policy | -0.000 | (-.022, .022) | -0.005 | (-.016, .006) | 0.006 | (-.007, .020) |
| Provision of extra-curricular lunchtime physical activity | -0.007 | (-.040, .026) | 0.003 | (-.014, .021) | 0.002 | (-.019, .023) |
| School attitude | **-0.008** | **(-.022, .005)** | 0.004 | (-.003, .012) | 0.004 | (-.004, .012) |
| Compulsory outdoor break time (in good weather) | -0.007 | (-.031, .016) | 0.007 | (-.006, .020) | 0.002 | (-.012, .016) |
| Break time rules: screen use allowed | -0.011 | (-.039, .016) | 0.001 | (-.014, .017) | 0.009 | (-.007, .026) |
| Break time rules: physically active activities allowed | 0.002 | (-.018, .022) | -0.006 | (-.016, .005) | 0.004* | (-.008, .017) |
| School (physical) environment | 0.001 | (-.002, .004) | -0.000 | (-.002, .002) | -0.001 | (-.003, .001) |

All exposure variables controlled for sex, age, BMI, and family SES; co-efficients shown in **bold** were taken forwards to multivariable models (p < .25). **^*^** Significant interaction with sex (and significant sub-group effect) were also taken forwards to multivariable models. **^ᴛ^** This was the only variable with p< .25 for LPA during the school day (p = .25), therefore no multivariable model was constructed for LPA.
