# Supplementary material for: School polices, programmes and facilities, and objectively measured sedentary time, LPA and MVPA: associations in secondary school and over the transition from primary to secondary school
Source: Int J Behav Nutr Phys Act. 2016 Apr 26;13:54. doi: 10.1186/s12966-016-0378-6 (PMC4845338; doi:10.1186/s12966-016-0378-6)
Supplement: Additional file 3: Table S3. — Simple models; Association of changes in the school environment with changes in activity intensity during the whole school day. (DOC 45 kb) [file 12966_2016_378_MOESM3_ESM.doc]

**Supplemental Table 3. Simple models; Association of changes in the school environment with changes in activity intensity during the whole school day.**

| Exposure | SED change | | LPA change | | MVPA change | |
| --- | --- | --- | --- | --- | --- | --- |
|  | β | (95% CI) | β | (95% CI) | 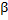 | (95% CI) |
| Length of break | **-0.07** | **(-0.15, 0.01)*** | 0.01 | (-0.03, 0.06) | **0.07** | **(0.03, 0.11)*†** |
| Total number of high quality facilities | -0.08 | (-0.54, 0.39) | -0.05 | (-0.31, 0.20) | 0.03 | (-0.23, 0.29) |
| Hours of PE  Stable (reference)  Decrease  Increase | -  **2.56**  0.49 | -  **(0.05, 5.07)***  (-1.71, 2.69) | -  **-1.56**  **-0.61** | -  **(-3.04, -0.07)***  **(-1.83, 0.62)†** | **-**  **-0.99**  0.33 | **-**  **(-2.22, 0.24)***  (-0.86, 1.52) |
| School has a physical activity policy  Stable (reference)  No/Yes  Yes/No | -  -1.57  0.50 | -  (-4.40, 1.25)  (-1.92, 2.93) | -  0.48  0.31 | -  (-1.09, 2.04)  (-0.88, 1.50) | -  0.29  **-0.90** | -  (-1.07, 1.65)  **(-2.26, 0.46)*** |
| Provision of extra-curricular lunchtime physical activity  Stable (reference)  No/Yes  Yes/No | -  **-2.16**  1.10 | -  **(-4.04, -0.28)***  (-2.75, 4.95) | -  0.61  -0.37 | -  (-0.53, 1.75)  (-2.57, 1.83) | -  **1.17**  -1.05 | -  **(0.30, 2.04)***  (-3.04, 0.94) |
| School attitude | 0.47 | (-1.14, 2.08) | **-0.51** | **(-1.34, 0.33)*** | 0.04 | (-0.80, 0.88) |
| Compulsory outdoor break (in good weather)  Stable (reference)  No/Yes  Yes/No | -  #  0.73 | -  (-1.41, 2.88) | -  #  -0.62 | -  (-1.79, 0.55) | -  #  -0.49 | -  (-1.68, 0.71) |
| Break time rules: screen use allowed  No/No (reference)  No/Yes  Yes/No  Yes/Yes | -  -0.82  #  -0.23 | -  (-3.72, 2.08)  -  (-4.10, 3.65) | -  -0.09  #  -0.31 | -  (-1.68, 1.51)  (-2.30, 1.69) | -  0.65  #  1.03 | -  (-1.00, 2.31)  (-1.07, 3.14) |
| Break time rules: physically active activities allowed  less / less (reference)  less / more  more / less  more / more | -  **1.91**  **1.87**  1.04 | -  **(-0.87, 4.69)***  **(-1.19, 4.93)***  (-1.75, 3.83) | -  **-1.27**  **-1.42**  **-1.24** | -  **(-2.74, 0.21)***  **(-3.13, 0.28)***  **(-2.67, 0.19)*** | -  -0.20  -0.37  **0.81** | -  (-1.74, 1.34)  (-1.84, 1.11)  **(-0.75, 2.36)†** |
| School (physical) environment | -0.01 | (-0.22, 0.19) | **0.08** | **(-0.03, 0.19)*** | **-0.07** | **(-0.18, 0.03)*** |

LPA, light physical activity; MVPA, moderate to vigorous physical activity; PE, physical education; PA, physical activity

β = beta coefficient; 95% CI = 95% confidence interval; # = coefficient not estimated due to small cell size (n=1)

Models adjusted for age, sex, BMI, socio-economic position and baseline value of the outcome variable. Exposures reflect change from baseline to follow up.

* = p<0.25**; †** = evidence of interaction with sex (as described in methods)**.** Variables in **bold** were included in a multivariable model.
