# Supplementary material for: School polices, programmes and facilities, and objectively measured sedentary time, LPA and MVPA: associations in secondary school and over the transition from primary to secondary school
Source: Int J Behav Nutr Phys Act. 2016 Apr 26;13:54. doi: 10.1186/s12966-016-0378-6 (PMC4845338; doi:10.1186/s12966-016-0378-6)
Supplement: Additional file 4: Table S4. — Simple models; Association of changes in the school environment with changes in activity intensity during lunchtime. (DOC 44 kb) [file 12966_2016_378_MOESM4_ESM.doc]

**Supplemental Table 4. Simple models; Association of changes in the school environment with changes in activity intensity during lunchtime.**

| Exposure | SED change | | LPA change | | MVPA change | |
| --- | --- | --- | --- | --- | --- | --- |
|  | β | (95% CI) | β | (95% CI) | 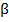 | (95% CI) |
| Length of break | **0.11** | **(-0.07, 0.29)*** | **-0.06** | **(-0.16, 0.04)*** | -0.03 | (-0.13, 0.06) |
| Total number of high quality facilities | 0.25 | (-0.87, 1.36) | -0.07 | (-0.70, 0.56) | -0.12 | (-0.71, 0.47) |
| Hours of PE  Stable (reference)  Decrease  Increase | -  2.53  -0.66 | -  (-2.86, 7.93)  (-5.73, 4.40) | -  -1.65  -0.96 | -  (-5.00, 1.70)  (-3.96, 2.04) | **-**  **-1.72**  1.49 | **-**  **(-4.57, 1.12)***  (-1.13, 4.12) |
| PA policy  Stable (reference)  No/Yes  Yes/No | -  **-4.02**  1.30 | -  **(-9.23, 1.20)***  (-4.23, 6.83) | -  **2.37**  0.19 | -  **(-0.75, 5.48)***  (-2.91, 3.29) | -  1.57  -1.26 | -  (-1.29, 4.43)  (-4.14, 1.61) |
| Provision of extra-curricular lunchtime PA  Stable (reference)  No/Yes  Yes/No | -  **-5.74**  **-8.49** | -  **(-9.37, -2.11)***  **(-16.72, -0.25)*** | -  **2.45**  **5.82** | -  **(0.32, 4.58)***  **(1.12, 10.53)*** | -  **3.31**  2.57 | -  **(1.32, 5.30)***  (-1.81, 7.0) |
| School attitude | -0.05 | (-3.39, 3.29) | 0.03 | (-1.95, 2.01) | 0.35 | (-1.43, 2.12) |
| Compulsory outdoor break (in good weather)  Stable (reference)  No/Yes  Yes/No | -  #  **4.51** | -  **(-0.49, 9.51)*** | -  #  **-2.41** | -  **(-5.21, 0.40)*** | -  #  **-2.00** | -  **(-4.65, 0.65)*** |
| Break time rules: screen use allowed  No/No (reference)  No/Yes  Yes/No  Yes/Yes | -  3.31  #  4.73 | -  (-3.79, 10.42)  (-3.96, 13.42) | -  **-2.43**  #  **-3.86** | -  **(-6.25, 1.39)***  **(-8.59, 0.88)*** | -  -1.20  #  -1.98 | -  (-4.94, 2.54)  (-6.59, 2.64) |
| Break time rules: physically active activities allowed  less / less (reference)  less / more  more / less  more / more | -  -1.69  -1.43  -1.35 | -  (-8.06, 4.68)  (-7.48, 4.63)  (-7.56, 4.86) | -  0.04  0.06  -0.48 | -  (-3.56, 3.63)  (-3.54, 3.66)  (-3.99, 3.03) | -  **1.55**  0.99  1.41 | -  **(-1.79, 4.89)†**  (-2.29, 4.27)  (-1.85, 4.66) |
| School (physical) environment | 0.09 | (-0.34, 0.52) | 0.05 | (-0.20, 0.31) | **-0.14** | **(-0.37, 0.09)*** |

LPA, light physical activity; MVPA, moderate to vigorous physical activity; PE, physical education; PA, physical activity

β = beta coefficient; 95% CI = 95% confidence interval; # = coefficient not estimated due to small cell size (n=1)

Models adjusted for age, sex, BMI, socio-economic position and baseline value of the outcome variable. Exposures reflect change from baseline to follow up.

* = p<0.25**; †** = evidence of interaction with sex (as described in methods)**.** Variables in **bold** were included in a multivariable model
